# Follow-up of COVID-19 recovered patients with mild disease

**DOI:** 10.1038/s41598-021-92717-8

**Published:** 2021-06-28

**Authors:** Alina Kashif, Manahil Chaudhry, Tehreem Fayyaz, Mohammad Abdullah, Ayesha Malik, Javairia Manal Akmal Anwer, Syed Hashim Ali Inam, Tehreem Fatima, Noreena Iqbal, Khadija Shoaib

**Affiliations:** 1 Medicine, CMH Lahore Medical College & Institute of Dentistry, Lahore, Pakistan; 2 Medicine, Hameed Latif Hospital, Lahore, Pakistan; 3 Medicine, Milton Keynes University Hospital, Milton Keynes, UK

**Keywords:** Epidemiology, Infectious diseases

## Abstract

COVID-19 may manifest as mild, moderate or severe disease with each grade of severity having its own features and post-viral implications. With the rising burden of the pandemic, it is vital to identify not only active disease but any post-recovery complications as well. This study was conducted with the aim of identifying the presence of post-viral symptomatology in patients recovered from mild COVID-19 disease. Presence or absence of 11 post-viral symptoms was recorded and we found that 8 of the 11 studied symptoms were notably more prevalent amongst the female sample population. Our results validate the presence of prolonged symptoms months after recovery from mild COVID-19 disease, particularly in association with the female gender. Hence, proving the post-COVID syndrome is a recognizable diagnosis in the bigger context of the post-viral fatigue syndrome.

## Introduction

The SARS-CoV-2 virus has led to a global health crisis ever since the first case of COVID-19 was reported in November 2012 in Wuhan, China^1^. COVID-19 primarily targets the respiratory system with variable initial symptoms including fever, sore throat, flu-like illness, and diarrhea^2^. There is a chance that some symptoms may linger even after the convalescence phase has subsided. The presence of symptoms after recovery from a viral disease is *broadly* recognized as a post-viral syndrome^3^. To date, various viruses have been associated with this syndrome, including the Coxsackie B virus, Epstein Bar virus, Influenza virus, *Candida albicans, Borrelia burgdorferi*, Enterovirus, Cytomegalovirus, Human Herpesvirus, Retrovirus, Borna virus, and Hepatitis C virus (HCV)^4^. The SARS outbreak in 2003 was due to a virus from the same RNA virus family as SARS-CoV-2, which has an established correlation with the development of several lingering symptoms after recovery from the acute infection^5^. Some reported symptoms include, malaise, myalgias, lack of concentration, and sleep disturbances.

A 131 patient based study in Wuhan revealed that 29% of their patients had persistence of symptoms after a 2-week post-discharge follow up^6^. This is one excerpt that provides evidence of this syndrome being present in COVID-19 patients; hence, we aimed to validate it further. With the third wave of COVID-19 pandemic rising in several parts of the world on a background of possible prolonged symptoms in previously affected patients, the disease burden will only increase. Hence, it is important to recognize not just active disease but the prevailing effects of prior disease in recovered patients as well.

## Materials and methods

The study participants were patients who accessed the health services at our hospital with mild COVID-19 disease. These patients were either admitted and discharged, or advised home quarantine to begin with. While consecutively including patients in the study, we confined our sample to those who presented with mild COVID-19 disease only, confirmed with a positive reverse transcriptase-polymerase chain reaction (RT-PCR) for SARS-CoV-2 virus (from a nasopharyngeal or throat swab) between April and June, 2020. Mild disease was classified based on the World Health Organization (WHO) guidelines^7^, defined as:

1. Mild clinical symptoms, i.e. fever < 38 °C (quelled without treatment).
2. With or without cough (no dyspnea, no gasping, no underlying chronic lung disease).
3. No imaging findings of pneumonia.

All patients between the ages of 18 to 65 years, suited to the aforementioned inclusion criteria who consented to participate were enlisted in an electronic patient record, along with their demographic and admission details. The patients were invited for a telephonic follow-up 3 months after being discharged from the hospital or three months after their hospital visit, for patients who were advised home quarantine initially.

Patients with moderate, severe, or critical COVID-19 disease, or those that required intensive care during their admission were excluded from the study. A chest X-ray (CXR) was done for all patients and those having any infiltrates suggestive of pulmonary involvement by SARS-CoV-2 were excluded. Other features that lead to the exclusion were, patients with missing contact details, those lost to follow up, being in-patient at the time of follow-up, and those who failed to give consent to participate when contacted for follow-up. Furthermore, patients who were diagnosed cases of angina, sleep disorders, psychiatric disorders, chronic fatigue syndromes, and neuromuscular disorders were also excluded, along with those who had cognitive or communication impairment that made the telephonic conversation difficult. All participants were also inquired whether they had contracted any other infections during the 3-month window. All patients with concurrent medical illnesses were excluded as well.

A total of 266 patients were contacted, out of which 26 could not be reached out to or were excluded because of the aforementioned reasons. 242 COVID-recovered patients were included in the final sample. Each person was then questioned about the demographic and admission details which were cross-checked with the electronic record to confirm their identity. They were asked open-ended questions regarding their health and feeling of any sort of symptom to identify the general self-reported health status before any formal questioning.

The presence or absence of the following post-viral symptoms was asked and noted: cough, persistent nausea/vomiting, headache, sleep disturbances (such as insomnia and unrefreshing sleep), decreased appetite, chest pain (chest discomfort that did not classify as angina, i.e. did not have the following signs: substernal discomfort of characteristic quality and duration, provoked by exertion or emotional stress and relieved by rest/nitrates), low mood, dizziness, palpitations, myalgias, and fatigability. This list of symptoms was designed after careful review of previous literature on symptoms associated or attributed to post-viral syndrome.

A formal approval from Combined Military Hospital Lahore Ethical Review Board (ERC# 251/2020) was sought prior to initiation of the study and informed consent was taken from all patients. The study was conducted in lines with the principles laid down in the Declaration of Helsinki. Demographic data and comorbidities were recorded as frequency and percentages. Chi-square test was used for comparison between nominal variables. Z-test was used to compare differences between ages. The data was analyzed using SPSSv25.

## Results

In total, 242 patients participated in the study, out of which 168 (69.4%) were males and 74 (30.6%) were females. Altogether, the presence of 11 post-viral symptoms was studied. The frequency and percentage of these symptoms is shown in Table 1.

**Table 1:** Frequency and percentages of various post-COVID symptoms in descending order of prevalence.

Symptom	Frequency	Percentage
Fatigability	101	41.7
Myalgias	85	35.1
Decreased appetite	58	24.0
Sleep disturbances	51	21.1
Headache	46	19.0
Low mood	44	18.2
Dizziness	35	14.5
Palpitation	30	12.4
Nausea/vomiting	29	12.0
Chest pain	26	10.7
Cough	16	6.6

The sample was stratified into two groups, those without any co-morbidity and those with co-morbidities present. The studied co-morbidities included diabetes mellitus (DM), hypertension (HTN), and ischemic heart disease (IHD).

Overall, only 29 people reported having one or more comorbidities while the rest of the 213 patients had none, as shown in Table 2. It was also noted that the group of people with comorbid conditions had a mean age of 50.23 ± 12.04 years as compared to the group having no co-morbidities with an average age of 33.65 ± 11.29 years (p < 0.00001). Two of the symptoms were significantly more prevalent amongst the group with co-morbidities, namely, decreased appetite (p = 0.023) and sleep disturbances (p = 0.004).

**Table 2:** Comparison of symptomatology amongst patients with and without co-morbidities.

	Co-morbidities (n = 29)		No co-morbidities (n = 213)		p-value
	Frequency	Percentage	Frequency	Percentage	
Fatigability	13	44.8	88	41.3	0.647
Myalgias	13	44.8	72	33.8	0.223
Palpitation	1	3.4	29	13.6	0.119
Dizziness	4	13.8	31	14.6	0.913
Low mood	8	27.6	36	16.9	0.162
Chest pain	2	6.9	24	11.3	0.476
Decreased appetite	12	41.4	46	21.6	**0.0230**
Sleep disturbances	12	41.4	39	18.3	**0.00427**
Headache	8	27.6	38	17.8	0.210
Nausea/vomiting	5	17.2	24	11.3	0.690
Cough	2	6.9	14	6.6	0.948

Bold values denote statistical significance at p ≤ 0.05

Analysis and comparison of symptomatology in accordance with gender was carried out as shown in Table 3. It depicts that 8 of the 11 studied post-viral symptoms had a significantly greater occurrence amongst females as compared to males. These included myalgias, decreased appetite, headache, low mood, nausea/vomiting, chest pain, sleep disturbances, and fatigability. While symptoms including palpitations, cough, and dizziness had no significant variation with gender.

**Table 3:** Differences in symptomatology amongst males and female participants.

	Male (n = 168)	Female (n = 74)	p-value
Myalgias	44 (26%)	41 (55%)	** < 0.00001**
Decreased appetite	28 (17%)	30 (41%)	**0.000051**
Sleep disturbances	27 (16%)	24 (32%)	**0.00435**
Headache	23 (14%)	23 (31%)	**0.00161**
Low mood	23 (14%)	21 (28%)	**0.00575**
Fatigability	63 (38%)	38 (51%)	**0.039**
Chest pain	11 (7%)	15 (20%)	**0.00137**
Nausea/vomiting	14 (8%)	15 (20%)	**0.00889**
Dizziness	23 (14%)	12 (16%)	0.0213
Palpitation	21 (13%)	9 (12%)	1.00
Cough	12 (7%)	4 (5%)	0.609

Bold values denote statistical significance at p ≤ 0.05

## Discussion

While Halpin et al.^8^ referred to them as “post-discharge” symptoms in a COVID-19 follow-up study, Islam et al. broadly described them as “post-viral fatigue” symptoms^9^. We aimed to infer whether these symptoms, which include, malaise, myalgias, lack of concentration, sleep disturbances, and others, were present following infection with COVID-19 disease. Existing literature has described the post-viral fatigue in moderate to severe cases of coronavirus recovered patients, it was unclear whether mild cases exhibited similar sequelae. One of the reasons from the rationale of studying mild cases only, was that Demeco et al.^10^ reported that in the majority, 81% of patients, COVID confers a mild disease. In addition, Garg et al.^11^ suggested in an editorial that a subgroup analysis of only mild COVID-19 patients would provide more insight to the post-viral fatigue syndrome as there is a scarce chance of chronic organ impairment in these cases. Therefore, in this study we aimed to identify residual symptoms in patients who recovered from mild COVID-19 disease due to infection by the SARS-CoV-2 virus.

Though a number of studies are still underway, and no consensus has been reached regarding the cut-off for how long the post-viral symptoms persist, following are some notable reported narratives. A study in Wuhan of 131 patients found that 29% of their patients had persistence of symptoms at a two week follow up^6^. Carfi et al.^12^ reported that, 87.4% recovered patients had persistence of at least one symptom, particularly fatigue and dyspnea at an average of 48 days post-discharge. In a survey led by the center for disease control and prevention (CDC) COVID-19 response team, amongst symptomatic adults with mild disease patients, 35% of the participants had not returned to their usual baseline health at 14–21 days after testing positive, with 47% amongst these being ≥ 50-years old^13^.

Halpin et al.^8^ observed that fatigue was the most commonly occurring symptom during follow-up for both ward (60.3%) and ICU (72%) patients, followed by respiratory symptoms (shortness of breath), while in our study, respiratory sequelae (cough and chest pain) were the least prevalent. It should be taken into consideration that moderate to severe cases of coronavirus can result in diffuse lung injury, pulmonary infiltrates along with respiratory distress. Furthermore, patients in ICU settings may require mechanical ventilation secondary to acute respiratory distress syndrome (ARDS) and pulmonary fibrosis. It can be inferred that persistence of respiratory symptoms seen in such cases may stem from long-term lung injury associated with more severe forms of the disease and hence, were not observed in our cohort.

A notable result from our study was the occurrence of sleep disturbances in 18.3% of healthy and in 41.4% of patients with existing co-morbidities, which was a statically significant finding (p = 0.00427). This renders that a strong correlation exists between disordered sleep and the presence of co-morbidities in patients recovering from COVID-19 infection. Altered sleep patterns have previously been reported by Moldofsky et al.^5^ who documented the presence of non-restorative, unrefreshing sleep for as long as 19.5 months in patients suffering from the chronic post-SARS syndrome (2003). Another study by Xiong et al. reported 17.7% of their COVID-19 survivor cohort had sleep disorders^14^. This goes to show that disordered sleep is a commonly occurring consequence of COVID-19 infection. This can be owing to isolation during lockdown, social distancing from family and friends and phobias associated with the pandemic.

There was an evident association of majority of the symptoms, specifically, decreased appetite, chest pain, headache, low mood, sleep disturbances, fatigability, myalgias, and nausea/vomiting with female gender. Similarly, Xiong et al.^14^ related physical decline/fatigue, post-activity polypnea and alopecia as being significantly more common in female survivors. Thus, an association of post-COVID sequelae with gender exists and demands further research.

We acknowledge some shortcomings in our study. Ours was a single-center study with a small sample size and the findings weren’t compared with a control group. Moreover, there always exists some element of recall bias with follow-up studies. Although, in more severe forms of the disease, the occurrence of the cytokine storm has proven to be a predictor of chronic fatigue syndrome (CFS) like symptoms in COVID-19 survivors, whereas, for less severe forms of the disease, there is still a need to study the pathogenesis leading to the occurrence of the post-COVID syndrome which cannot be explained entirely by the cytokine storm. Lastly, it is important to include various ethnicities and socio-demographically diverse groups in study samples to study the association of the post-COVID syndrome with the ethnicity.

## Conclusion

We reported various post-viral sequelae, the most common being fatigue, in mild-COVID-19 recovered patients 3 months after being discharged. The existence of post-COVID recovery symptoms warrants recognition of COVID-19 as a cause of post-viral fatigue syndrome, even in mild forms of the disease. This can help clinicians to plan arrangements such as follow-up visits, rehabilitation, cognitive behavioral therapy, and even simple measures like incorporation of counseling sessions at discharge to reduce patient anxiety regarding extended symptoms.
